# C1q CIC in Lupus Nephritis: An Analysis of 883 Patients

**DOI:** 10.7150/ijms.125522

**Published:** 2026-07-01

**Authors:** Huang-Chen Chang, Yen-Ching Wu, Jun-Peng Chen, Wen-Nan Huang, Yi-Hsing Chen, Yi-Ming Chen

**Affiliations:** 1 Division of Allergy, Immunology and Rheumatology, Taichung Veterans General Hospital, Taichung, Taiwan.; 2 Department of Medical Research, Taichung Veterans General Hospital, Taichung, Taiwan.; 3 Center of Rheumatology and Immunology, China Medical University Hospital, Taichung, Taiwan.; 4 Graduate Institute of Clinical Medicine, National Chung Hsing University, Taichung, Taiwan.; 5 Department of Post-Baccalaureate Medicine, College of Medicine, National Chung Hsing University, Taichung, Taiwan.

**Keywords:** systemic lupus erythematosus, lupus nephritis, C1q CIC, rule-out biomarker

## Abstract

**Objectives:**

Lupus nephritis (LN) is a major complication of systemic lupus erythematosus (SLE). This study evaluated the association of C1q-circulating immune complexes (C1q CIC) with LN and their potential adjunctive rule-out value compared with conventional biomarkers.

**Methods:**

We conducted a retrospective analysis of 883 SLE patients from the Asia Pacific Lupus Collaboration (APLC) cohort in Taiwan. C1q CIC was assessed for its association with disease activity and compared to anti-dsDNA, C3, and C4. Logistic regression and ROC analysis were performed.

**Results:**

C1q CIC levels were significantly elevated in active SLE and in non-renal manifestations, including cutaneous involvement, serositis, and hematologic abnormalities. In patients with LN, C1q CIC levels were significantly higher than in those without LN, whereas anti-dsDNA, C3, and C4 showed no significant differences. Overall discriminative performance was modest, and C1q CIC is not suitable as a diagnostic or screening tool for LN. At a cutoff of 5.31 μg Eq/mL, C1q CIC showed a high negative predictive value (NPV = 86.04%) but low positive predictive value (PPV = 29.51%). In stratified analysis, discriminative ability was limited in patients with low disease activity (SLEDAI-2K < 4; AUC = 0.55), whereas NPV remained high (97.15%).

**Conclusion:**

C1q CIC may serve as an adjunctive rule-out biomarker for LN, particularly when levels are low or in patients with low disease activity. Although not suitable for diagnosis or screening, its relatively high NPV may help identify patients at lower risk of LN. Results should be interpreted in conjunction with clinical and laboratory assessment.

## Introduction

Systemic lupus erythematosus (SLE) is a complex autoimmune disorder marked by the generation of autoantibodies, activation of complement, and deposition of immune complexes, all of which led to inflammation across multiple systems and damage to tissues 1, 2. Although SLE presents with a variety of clinical symptoms, lupus nephritis (LN) remains one of its most serious and life-threatening complications 3. Research indicates that 31-48% of individuals with SLE develop LN, with most cases arising within five years of the initial diagnosis 4. This kidney involvement has a significant impact on long-term outcomes and is linked to higher rates of morbidity and mortality 5. Approximately 10-30% of LN patients progress to end-stage renal disease (ESRD) within a decade 6-8. Consequently, swift diagnosis and timely treatment of renal flares are essential for enhancing outcomes in LN 9, 10.

Conventional biomarkers used to monitor LN, including anti-double stranded DNA antibodies (anti-dsDNA), complement C3, and C4 are essential for assessing disease activity 11-13. However, these biomarkers have significant limitations, as complement levels and anti-dsDNA titers do not consistently correlate with pathologic processes 14. Additionally, inconsistencies across studies highlight the limitations and lack of consensus regarding their predictive value for LN 15, highlighting the need for additional adjunctive biomarkers.

C1q-circulating immune complexes (C1q CIC) have recently gained attention as a potential adjunctive biomarker associated with immune complex-mediated disease activity in SLE and LN 16-20. C1q CIC is essential for immune complex clearance and complement activation. In SLE, impaired clearance leads to the accumulation of C1q CIC, which has been closely linked to active nephritis and glomerular inflammation 21, 22. Nevertheless, despite these pathophysiological links, the precise clinical utility of C1q CIC remains incompletely defined. Although previous studies have compared it with conventional serological markers (anti-dsDNA, C3, and C4), inconsistencies persist regarding its additive value, particularly across different levels of disease activity 20.

The present study aimed to examine the relationship between C1q CIC levels and SLE disease activity, to compare its performance with conventional markers, and evaluate its potential role in LN risk assessment across different levels of disease activity.

## Methods

### Study Participants

This retrospective study examined 883 patients diagnosed with systemic lupus erythematosus (SLE) from the Asia Pacific Lupus Collaboration (APLC) cohort at a tertiary medical center in Taiwan, covering the years 2017 to 2021 23, 24, as previously detailed. All participants fulfilled either the 1997 American College of Rheumatology (ACR) criteria or the 2012 Systemic Lupus International Collaborating Clinics (SLICC) criteria for SLE 25-27. The study protocol received approval from the Institutional Review Board of Taichung Veterans General Hospital (IRB No. CF21176A).

### Study Design

Patients were categorized as C1q CIC-positive (≥10.8 μg Eq/mL) or C1q CIC-negative (<10.8 μg Eq/mL). Demographic characteristics, clinical features, laboratory findings, and medication use within six months of SLE diagnosis were compared between the two groups. Disease activity was evaluated using the SLE Disease Activity Index 2000 (SLEDAI-2K) 28, 29, with scores ≥4 indicating active disease 30. Patients were also stratified by disease activity, and the same variables were compared between groups. The relationships between C1q CIC and conventional biomarkers (anti-dsDNA, C3, and C4) were examined. LN was defined as a urine protein-to-creatinine ratio (UPCR) ≥500 mg/g, and biomarker levels were compared between patients with and without LN. Logistic regression analysis was conducted to identify independent predictors of LN, and receiver operating characteristic (ROC) curve analysis was performed to determine the cutoff with the best discriminative performance.

### Measurement of CIC

C1q CIC was measured using the QUANTA Lite C1q CIC ELISA (INOVA Diagnostics, USA). Microwells are pre-coated with purified human C1q to capture circulating immune complexes capable of binding C1q. After incubation and washing, bound complexes were detected using a peroxidase-labeled anti-human IgG conjugate with TMB substrate. Absorbance was measured at 450 nm, and concentrations were derived from the calibration curve and expressed in μg Eq/mL. According to the manufacturer's instructions, values ≥10.8 μg Eq/mL were considered positive.

### Clinical and Laboratory Data

Clinical and laboratory parameters included hematologic, biochemical, immunologic data, and urinalysis. All assays were performed according to the respective manufacturers' instructions. Complement C3 and C4 levels were measured by nephelometry (Beckman Coulter DxC 700 AU, CA, USA). Hypocomplementemia was defined as C3 < 87 mg/dL and C4 < 19 mg/dL. Anti-dsDNA was measured using ELISA (Inova Diagnostics Inc., CA, USA), with a cutoff of ≥139 WHO units/mL defined as positive.

### Medication Use

We also evaluated the influence of medications by analyzing whether patients had received any of the following within six months prior to data collection: prednisolone, hydroxychloroquine, methotrexate, azathioprine, mycophenolate mofetil, mycophenolic acid, or cyclosporine.

### Statistical Analysis

Continuous variables (e.g., age, hemoglobin, platelet count) were reported as medians with interquartile ranges (IQR), while categorical variables (e.g., low C3, low C4, anti-dsDNA antibody positivity, and C1q CIC positivity) were presented as frequencies and percentages, as defined according to the manufacturer's instructions. Group comparisons were made using the chi-square test for categorical data and the Mann-Whitney U test for continuous data. Multivariate logistic regression was employed to determine predictors of LN, with results shown as odds ratios (ORs) and 95% confidence intervals (CIs). The model was adjusted for age, hypertension, diabetes mellitus, hemoglobin, eGFR, low C4, and C1q CIC positivity. The discriminative performance of C1q CIC in patients with and without LN was assessed using ROC curve analysis, and the optimal cutoff value was determined using the Youden index. An exploratory ROC analysis incorporating C1q CIC, hemoglobin, age, sex, and anti-dsDNA was additionally performed to evaluate the discriminative performance of a combined model with C1q CIC and clinical variables. Stratified analyses according to disease activity (SLEDAI-2K < 4 vs. ≥ 4) were further conducted to assess whether the performance of C1q CIC varied across different activity states. Relationships between variables were examined using scatterplots and Spearman correlation coefficients. A two-tailed *p* value of less than 0.05 was deemed statistically significant. All statistical analyses were performed using SPSS version 23.0 (IBM Corp., Armonk, NY, USA).

## Results

### Comparison of Characteristics Between C1q CIC-Positive and -Negative Patients

Our findings indicated that patients who tested positive for C1q CIC were younger, with a mean age of 38.8 years, and experienced the onset of SLE at an earlier age. Clinically, patients presenting with non-scarring alopecia, renal involvement, and leukopenia showed significantly higher rates of C1q CIC positivity (*p* < 0.05). Laboratory findings revealed that C1q CIC-positive patients had lower hemoglobin levels, decreased complement levels, higher positivity for anti-dsDNA, and higher SLEDAI-2K scores (Table 1). The proportion of patients with SLEDAI-2K ≥ 4 was substantially higher in C1q CIC-positive patients (*p* < 0.001). Diabetes mellitus was less frequent in C1q CIC-positive patients. C1q CIC-positive patients also had significantly higher UPCR (p < 0.001), consistent with greater renal involvement.

### Comparison of Characteristics Between Patients With and Without LN

Patients with and without LN were compared as shown in Table 2. The LN group showed markedly higher rates of renal disorder by SLICC criteria (90.0% vs. 36.6%, *p* < 0.001), lower hemoglobin, lower eGFR, and substantially elevated UPCR. C1q CIC positivity was more prevalent in LN patients (32.4% vs. 21.0%,* p* = 0.002). Disease activity was considerably higher in the LN group, with 90.6% of patients having SLEDAI-2K ≥ 4 compared with 34.0% in the non-LN group (*p* < 0.001). Diabetes mellitus was more frequent in LN patients (16.5% vs. 10.3%, *p* = 0.024).

### C1q CIC in SLE Disease Activity and Its Association with Conventional Biomarkers

**Figure 1:**
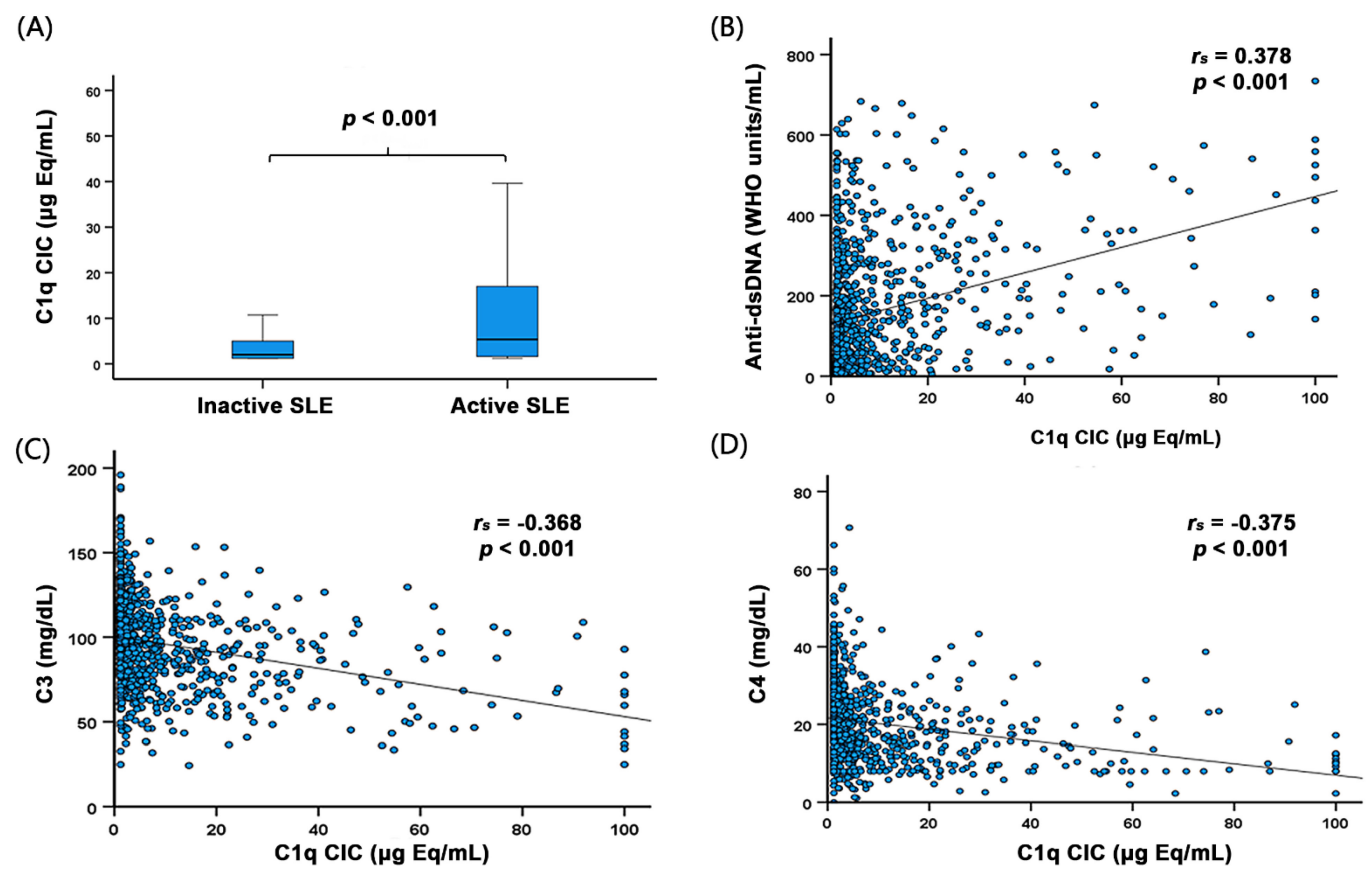
C1q CIC levels were elevated in active SLE and showed moderate correlations with anti-dsDNA, C3, and C4.

Patients with active SLE showed notably elevated levels of C1q CIC compared to those with inactive disease (*p* < 0.001) (Figure 1A), indicating its association with SLE disease activity. Additionally, levels of C1q CIC exhibited a positive relationship with anti-dsDNA antibodies (*r_s_* = 0.378, *p* < 0.001) and negative correlations with C3 and C4 (*r_s_* = -0.368 and -0.375, *p* < 0.001, respectively) (Figure 1B-D), indicating modest associations with conventional biomarkers. Stratified analysis according to disease activity showed that in patients with higher disease activity (SLEDAI-2K ≥ 4), the AUC was 0.687 (*p* < 0.001) with a negative predictive value (NPV) of 75.72%. In contrast, in patients with low disease activity (SLEDAI-2K < 4), the AUC was 0.55 (*p* = 0.566), with a higher NPV of 97.15%.

### C1q CIC in Patients With and Without LN

**Figure 2:**
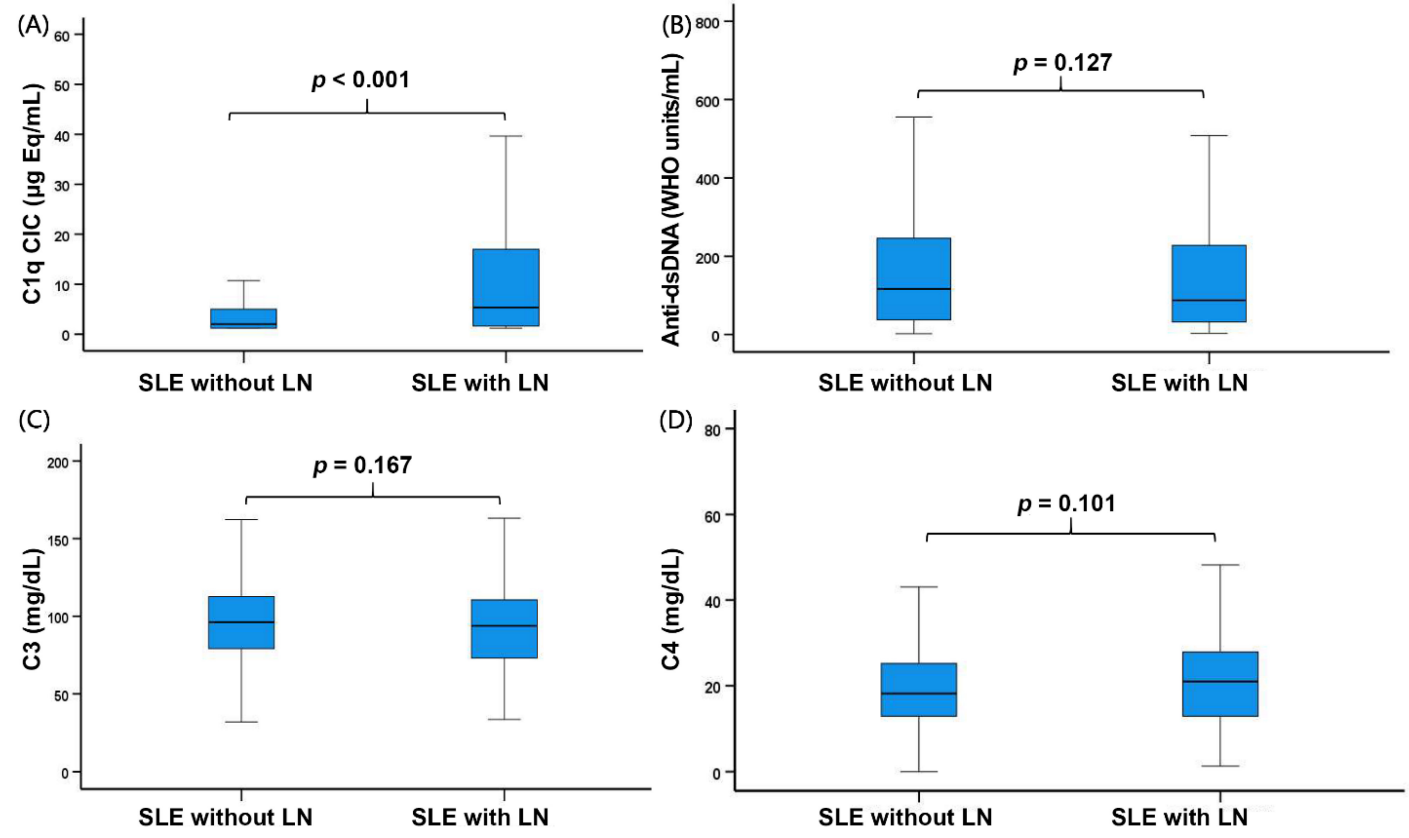
C1q CIC levels were significantly higher in LN patients (*p* < 0.001), while anti-dsDNA, C3 and C4 levels showed no significant differences.

**Figure 3:**
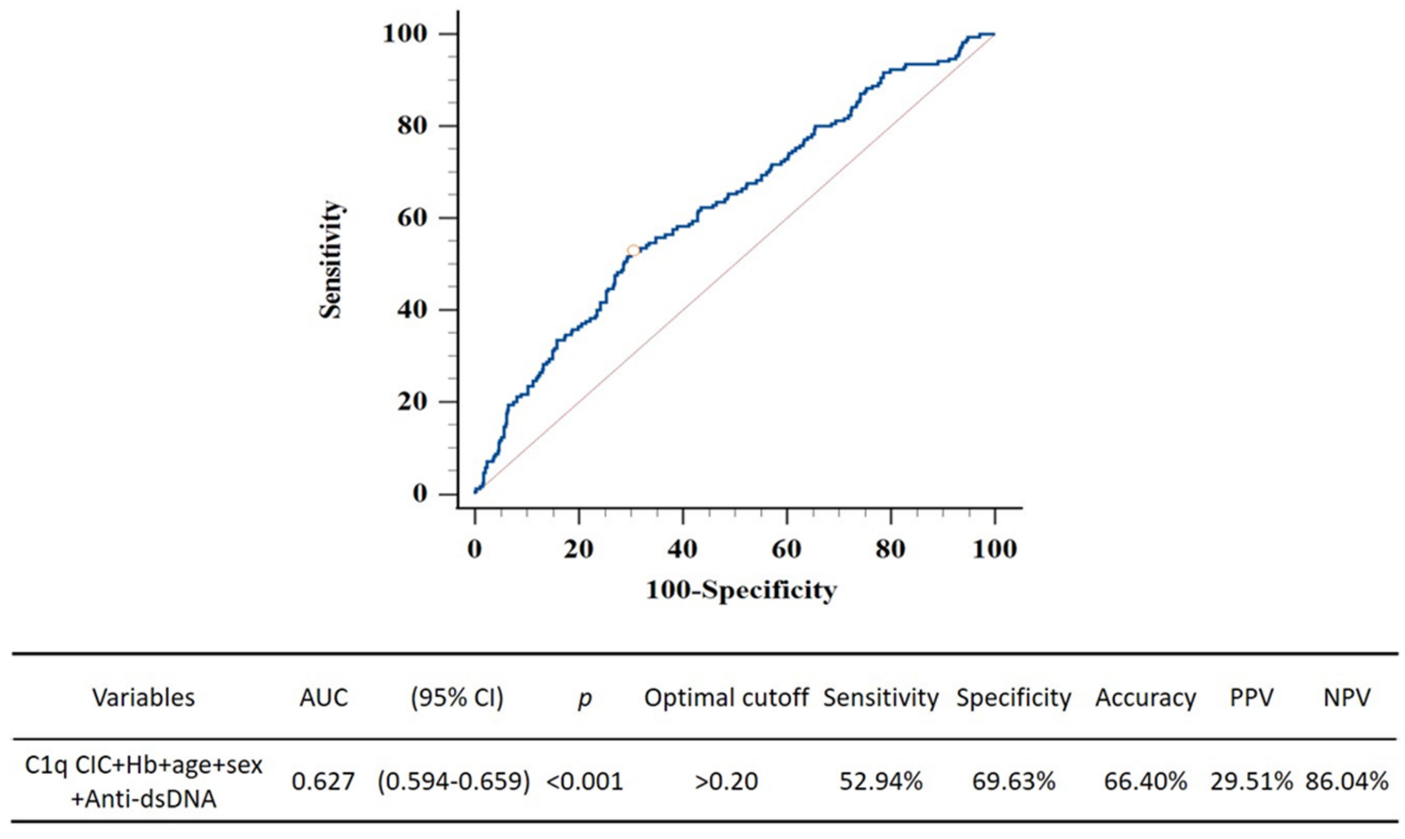
ROC curve of the combined biomarker model in patients with and without LN. C1q CIC: Circulating immune complexes that bind C1q; Hb: hemoglobin; Anti-dsDNA: anti-double stranded DNA antibodies.

We compared the levels of C1q CIC, anti-dsDNA, C3, and C4 between patients with and without LN. Among these biomarkers, only C1q CIC levels were significantly elevated in the LN group compared to the non-LN group (*p* < 0.001), while no significant differences were observed for anti-dsDNA, C3, and C4 levels (Figure 2). Univariate logistic regression analysis identified several variables associated with LN, including age, hypertension, diabetes mellitus, SLEDAI-2K score, hemoglobin, eGFR, low C4, and C1q CIC positivity. In the multivariable model adjusted for age, hypertension, diabetes mellitus, hemoglobin, eGFR, low C4, and C1q CIC positivity, C1q CIC positivity remained an independent predictor of LN (OR = 1.87, 95% CI 1.22-2.86, *p* = 0.004; Table 3). The optimal cutoff value of 5.31 μg Eq/mL for C1q CIC was derived from the ROC curve using the Youden index. At this cutoff, C1q CIC demonstrated limited rule-in performance but relatively strong rule-out capacity. An exploratory ROC model combining C1q CIC, hemoglobin, age, sex, and anti-dsDNA demonstrated modest performance in LN discrimination (AUC 0.627; 95% CI 0.594-0.659) (Figure 3).

## Discussion

In the present cohort, C1q CIC demonstrated modest discriminative performance for LN (AUC = 0.627; 95% CI 0.594-0.659), which was numerically superior to the conventional combination of anti-dsDNA, C3, and C4 (AUC = 0.595) (data not shown). At the optimal cutoff of 5.31 μg Eq/mL determined by the Youden index, the NPV was relatively high (NPV = 86.04%), whereas the positive predictive value (PPV) remained low (PPV = 29.51%). These findings indicate that C1q CIC should not be used as a stand-alone marker for LN but may serve as an adjunctive rule-out tool, in combination with conventional markers, for identifying patients at lower risk of LN, particularly when levels are low.

In addition, stratified analysis according to disease activity revealed that the discriminative performance of C1q CIC was context-dependent. In patients with low disease activity, the discriminatory ability was limited (AUC = 0.55), although the NPV remained high (NPV = 97.15%). This reduced performance may be explained by the lower burden of immune complex formation and tissue deposition in quiescent states, resulting in insufficient differences in C1q CIC levels between patients with and without LN. Nevertheless, the persistently high NPV in this subgroup supports its potential adjunctive value for ruling out LN even in low-activity settings.

In our study, we found that serum C1q CIC levels were notably higher in patients with active SLE than in those with inactive disease, consistent with previous reports 31, 32. However, the correlation between C1q CIC and anti-dsDNA was only modest (*r*_s_ = 0.378), contrasting with the stronger associations reported by Shuhong Chi and E. Akhter (*r* = 0.796 and 0.69, respectively) 17, 33. Variability in C1q CIC and anti-dsDNA detection assays across studies may partly account for these discrepancies, as differences in commercial kit methodologies have been reported. To date, no comprehensive studies have compared C1q CIC assays from various manufacturers 34.

Previous studies have indicated elevated C1q CIC levels in patients with LN. Shuhong Chi *et al*. reported increased C1q CIC in LN, but did not directly compare it with conventional biomarkers 17. Xiaoying *et al*. demonstrated that C1q CIC had better sensitivity for LN than anti-dsDNA; however, their analysis lacked multivariate adjustment 36. In contrast, our study not only compared C1q CIC with traditional biomarkers (anti-dsDNA, C3, and C4), but also confirmed its role as an independent predictor of LN after adjusting for relevant clinical covariates. Importantly, we further observed that correlations between C1q CIC and anti-dsDNA (*r_s_* = 0.601, *p* < 0.001), C3 (*r_s_* = -0.570, *p* < 0.001), C4 (*r_s_* = -0.534, *p* < 0.001) were more pronounced in patients with LN (data not shown), as compared with those without LN, (anti-dsDNA, *r_s_* = 0.331, *p* < 0.001; C3, *r_s_* = -0.318, *p* < 0.001; C4, *r_s_*= -0.351, *p* < 0.001), suggesting a closer association with immune complex-mediated complement activation in this subgroup, although this likely reflects systemic immune activity rather than kidney-specific injury.

From a mechanistic perspective, C1q is an upstream component of the classical complement pathway that directly binds immune complexes, whereas C3 and C4 primarily reflect downstream complement consumption and are influenced by systemic factors, including hepatic synthesis, particularly in states of modest disease activity 37. In our cohort, C1q CIC levels were significantly elevated in LN, whereas anti-dsDNA, C3, and C4 showed no significant differences. We hypothesize that activation of the upstream classical complement pathway driven by immune complexes in LN may be more readily captured by C1q than by downstream markers such as C3 and C4. In addition, systemic factors may further influence the levels of C3 and C4, potentially limiting their predictive performance in this setting.

From a clinical perspective, the relatively high NPV supports the adjunctive value of C1q CIC in identifying patients at lower risk of LN when interpreted together with standard clinical and laboratory parameters. Given its modest overall discrimination and lack of renal specificity, however, C1q CIC should not be regarded as a confirmatory diagnostic tool. Renal biopsy remains the gold standard for the definitive diagnosis of LN 38.

This study has limitations. First, its retrospective, single-center design may introduce selection bias and limit generalizability. Second, the absence of renal biopsy data led us to define LN based on UPCR ≥500 mg/g, which reflects clinically significant proteinuria but cannot distinguish active nephritis from chronic damage or non-lupus causes. This approach may have contributed to the relatively high NPV and low PPV observed. Third, the relatively low disease activity in the cohort may have attenuated the observed associations, and the findings may not be readily extrapolated to patients with higher activity. Furthermore, elevated C1q CIC may result from extra-renal manifestations such as cutaneous, serosal, or hematologic involvement. Therefore, a comprehensive clinical and laboratory evaluation is required when interpreting C1q CIC results. Finally, the cross-sectional design precludes assessment of its prognostic value for long-term renal outcomes. Prospective studies with histological confirmation are warranted to validate these findings.

## Conclusion

C1q CIC may serve as an adjunctive rule-out biomarker for lupus nephritis, particularly when levels are low or in patients with low disease activity. However, elevated C1q CIC is not specific for renal involvement and may also reflect cutaneous, serosal, or hematologic disease activity. Given its modest discriminative performance and low PPV, C1q CIC should not be used as a stand-alone diagnostic tool but interpreted together with comprehensive clinical and laboratory assessment for lupus nephritis.

## Figures and Tables

**Table 1:** Demographic data of participants.

	C1q CIC Negative (n = 678)		C1q CIC Positive (n = 205)		*p* value
**Demographics**					
Age	46.1	(37.5-55.7)	38.8	(30.2-50.6)	<0.001**
Sex					0.031*
Female	620	(91.4%)	177	(86.3%)	
Male	58	(8.6%)	28	(13.7%)	
SLE Onset Age	32.0	(24.0-42.0)	27.0	(21.0-38.0)	0.002**
Smoking	36	(5.3%)	16	(7.8%)	0.184
Hypertension	232	(34.2%)	68	(33.2%)	0.781
Diabetes mellitus	87	(12.87%)	14	(6.8%)	0.018*
**SLICC Clinical Criteria**					
Acute cutaneous lupus	299	(44.1%)	99	(48.3%)	0.290
Chronic cutaneous lupus	83	(12.2%)	15	(7.3%)	0.049*
Oral or nasal ulcers	75	(11.1%)	27	(13.2%)	0.408
Non-scarring alopecia	40	(5.9%)	24	(11.7%)	0.005**
Synovitis involving two or more joints	401	(59.1%)	113	(55.1%)	0.306
Serositis	80	(11.8%)	35	(17.1%)	0.049*
Renal disorder	298	(44.0%)	118	(57.6%)	<0.001**
Neurologic disorder	61	(9.0%)	10	(4.9%)	0.057
Hemolytic anemia	58	(8.6%)	19	(9.3%)	0.751
Leukopenia < 4,000/mm3 or Lymphopenia <1,000/mm3	257	(37.9%)	99	(48.3%)	0.008**
Thrombocytopenia < 100,000/mm3	128	(18.9%)	40	(19.5%)	0.847
**Laboratory Examination**					
WBC (/μL)	5920.0	(4570.0-7700.0)	5440.0	(4230.0-7855.0)	0.163
Hb (g/dL)	12.8	(11.6-13.8)	12.3	(11.1-13.4)	<0.001**
Platelet x10^3^/μL	234.0	(188.8-284.0)	231.0	(186.0-287.3)	0.909
Creatinine (mg/dL)	0.7	(0.6-0.9)	0.7	(0.6-0.9)	0.445
eGFR (mL/min/1.73m^2^)	92.9	(74.0-108.1)	99.2	(78.8-117.4)	0.009**
UPCR (mg/g)	123.1	(66.7-298.2)	174.9	(94.7-638.7)	<0.001**
**Immunology**					
Low C3	449	(66.2%)	166	(81.0%)	<0.001**
Low C4	342	(50.4%)	143	(69.8%)	<0.001**
Anti-dsDNA positive	237	(35.0%)	144	(70.2%)	<0.001**
**SLEDAI-2K score**	2	(0-4)	4	(2-6)	<0.001**
SLEDAI-2K score group					<0.001**
SLEDAI-2K ≥ 4	258	(38.1%)	139	(67.8%)	
SLEDAI-2K < 4	420	(61.9%)	66	(32.2%)	
**Medication**					
Prednisolone	537	(79.2%)	182	(88.8%)	0.002**
Hydroxychloroquine	622	(91.7%)	184	(89.8%)	0.378
Methotrexate	36	(5.3%)	10	(4.9%)	0.807
Azathioprine	211	(31.1%)	65	(31.7%)	0.874
Mycophenolate mofetil	25	(3.7%)	24	(11.7%)	<0.001**
Mycophenolic acid	94	(13.9%)	29	(14.1%)	0.919
Cyclosporin	48	(7.1%)	20	(9.8%)	0.208

Chi-Square test or Mann-Whitney U test, Median (IQR). WBC: white blood cell; Hb: hemoglobin; eGFR: estimated glomerular filtration rate; UPCR: urine protein and creatinine ratio; Anti-dsDNA: anti-double stranded DNA antibodies; C1q CIC: circulating immune complexes that bind C1q.

**Table 2:** Comparison of demographic, clinical, and laboratory characteristics between patients with lupus nephritis (LN) and those without LN.

	without LN (n= 708)		LN (n= 170)		*p* value
**Demographics**					
Age	45.1	(36.8-54.5)	41.9	(31.7-53.5)	0.025*
Sex					0.211
Female	643	(90.8%)	149	(87.6%)	
Male	65	(9.2%)	21	(12.4%)	
SLE Onset Age	31.0	(23.0-42.0)	28.0	(22.8-39.3)	0.156
Smoking	41	(5.8%)	11	(6.5%)	0.736
Hypertension	197	(27.8%)	102	(60.0%)	<0.001**
Diabetes mellitus	73	(10.3%)	28	(16.5%)	0.024*
**SLICC Clinical Criteria**					
Acute cutaneous lupus	326	(46.0%)	68	(40.0%)	0.155
Chronic cutaneous lupus	80	(11.3%)	18	(10.6%)	0.791
Oral or nasal ulcers	82	(11.6%)	19	(11.2%)	0.882
Non-scarring alopecia	52	(7.3%)	11	(6.5%)	0.692
Synovitis involving two or more joints	414	(58.5%)	97	(57.1%)	0.737
Serositis	87	(12.3%)	28	(16.5%)	0.147
Renal disorder	259	(36.6%)	153	(90.0%)	<0.001**
Neurologic disorder	60	(8.5%)	10	(5.9%)	0.263
Hemolytic anemia	62	(8.8%)	14	(8.2%)	0.828
Leukopenia < 4,000/mm^3^ or Lymphopenia <1,000/mm^3^	291	(41.1%)	64	(37.6%)	0.410
Thrombocytopenia < 100,000/mm^3^	139	(19.7%)	28	(16.5%)	0.342
**Laboratory Examination**					
WBC (/μL)	5740.0	(4360.0-7390.0)	7095.0	(5025.0-9127.5)	<0.001**
Hb (g/dL)	12.8	(11.6-13.8)	12.1	(10.8-13.4)	<0.001**
Platelet x10^3^/μL	231.0	(188.5-282.0)	244.0	(186.5-300.0)	0.069
Creatinine (mg/dL)	0.7	(0.6-0.8)	0.9	(0.7-1.3)	<0.001**
eGFR (mL/min/1.73 m^2^)	96.0	(80.1-112.9)	75.9	(50.9-103.5)	<0.001**
UPCR (mg/g)	100.0	(61.1-182.5)	1038.0	(751.7-2135.5)	<0.001**
**Immunology**					
Low C3	485	(68.5%)	128	(75.3%)	0.083
Low C4	376	(53.1%)	107	(62.9%)	0.021*
Anti-dsDNA positive	312	(44.1%)	66	(38.8%)	0.215
C1q CIC					0.002**
Positive	149	(21.0%)	55	(32.4%)	
Negative	559	(79.0%)	115	(67.6%)	
**SLEDAI-2K score**	2	(0-4)	6	(4-8)	<0.001**
SLEDAI-2K score group					<0.001**
SLEDAI-2K ≥ 4	241	(34.0%)	154	(90.6%)	
SLEDAI-2K < 4	467	(66.0%)	16	(9.4%)	
**Medication**					
Prednisolone	557	(78.7%)	160	(94.1%)	<0.001**
Hydroxychloroquine	650	(91.8%)	153	(90.0%)	0.449
Methotrexate	41	(5.8%)	5	(2.9%)	0.134
Azathioprine	238	(33.6%)	38	(22.4%)	0.005**
Mycophenolate mofetil	33	(4.7%)	16	(9.4%)	0.015*
Mycophenolic acid	58	(8.2%)	65	(38.2%)	<0.001**
Cyclosporin	49	(6.9%)	19	(11.2%)	0.062

Chi-Square test or Mann-Whitney U test, Median (IQR). WBC: white blood cell; Hb: hemoglobin; eGFR: estimated glomerular filtration rate; UPCR: urine protein and creatinine ratio; Anti-dsDNA: anti-double stranded DNA antibodies; C1q CIC: circulating immune complexes that bind C1q.

**Table 3:** Logistic regression identifies biomarkers associated with LN in SLE patients.

	Univariate			Multivariable		
	OR	95% CI	p value	OR	95% CI	p value
Age	0.99	(0.97-1.00)	0.031*	0.96	(0.94-0.98)	<0.001**
Sex						
Female	Reference					
Male	1.39	(0.83-2.35)	0.213			
Hypertension	3.89	(2.75-5.51)	<0.001**	3.34	(2.26-4.95)	<0.001**
Diabetes mellitus	1.72	(1.07-2.75)	0.025*	1.43	(0.83-2.48)	0.200
SLEDAI-2K score	1.40	(1.31-1.49)	<0.001**			
Hb (g/dL)	0.87	(0.79-0.95)	0.002**	0.99	(0.89-1.10)	0.882
Platelet x103/μL	1.00	(1.00-1.00)	0.085			
eGFR (mL/min/1.73m2)	0.98	(0.97-0.98)	<0.001**	0.98	(0.97-0.98)	<0.001**
Low C3	1.40	(0.96-2.06)	0.084			
Low C4	1.50	(1.06-2.12)	0.021*	1.21	(0.82-1.79)	0.339
Anti-dsDNA positivity	1.00	(1.00-1.00)	0.665			
C1q CIC positivity	1.01	(1.01-1.02)	0.001**	1.87	(1.22-2.86)	0.004**

Logistic regression. *p < 0.05, **p < 0.01. The models included both continuous variables (age, hemoglobin, SLEDAI-2K score, platelet count, and eGFR) and categorical variables (hypertension, diabetes mellitus, low C3, low C4, anti-dsDNA positivity, and C1q CIC positivity, defined according to the manufacturer's recommended cutoff). The multivariable model was adjusted for age, hypertension, diabetes mellitus, hemoglobin, eGFR, low C4, and C1q CIC positivity. Anti-dsDNA: anti-double stranded DNA antibodies; C1q CIC: circulating immune complexes that bind C1q.
